# An In Vitro Model of Glioma Development

**DOI:** 10.3390/genes14050990

**Published:** 2023-04-27

**Authors:** Gabriella Schiera, Patrizia Cancemi, Carlo Maria Di Liegro, Flores Naselli, Sara Volpes, Ilenia Cruciata, Paola Sofia Cardinale, Fabiola Vaglica, Matteo Calligaris, Anna Paola Carreca, Roberto Chiarelli, Simone Dario Scilabra, Olga Leone, Fabio Caradonna, Italia Di Liegro

**Affiliations:** 1 Department of Biological, Chemical and Pharmaceutical Sciences and Technologies, University of Palermo, Viale delle Scienze, Edificio 16, 90128 Palermo, Italy; gabriella.schiera@unipa.it (G.S.); patrizia.cancemi@unipa.it (P.C.); carlomaria.diliegro@unipa.it (C.M.D.L.); flores.naselli@unipa.it (F.N.); sara.volpes@unipa.it (S.V.); ilenia.cruciata@unipa.it (I.C.); paola.sofia.cardinale@gmail.com (P.S.C.); fabiola.vaglica@unipa.it (F.V.); roberto.chiarelli@unipa.it (R.C.); 2 Proteomics Group, Department of Research, ISMETT-IRCCS, Ri.MED Foundation, 90127 Palermo, Italy; mcalligaris@fondazionerimed.com (M.C.); apcarreca@fondazionerimed.com (A.P.C.); sdscilabra@fondazionerimed.com (S.D.S.); 3 Department of Biomedicine, Neurosciences and Advanced Diagnostics, University of Palerm, Via del Vespro, 129, 90127 Palermo, Italy; olga.leonee98@gmail.com

**Keywords:** astrocytomas, astrocyte cell lines, epigenetic alterations, chromosome alterations, proteomics, metalloproteinases, extracellular vesicles (EVs)

## Abstract

Gliomas are the prevalent forms of brain cancer and derive from glial cells. Among them, astrocytomas are the most frequent. Astrocytes are fundamental for most brain functions, as they contribute to neuronal metabolism and neurotransmission. When they acquire cancer properties, their functions are altered, and, in addition, they start invading the brain parenchyma. Thus, a better knowledge of transformed astrocyte molecular properties is essential. With this aim, we previously developed rat astrocyte clones with increasing cancer properties. In this study, we used proteomic analysis to compare the most transformed clone (A-FC6) with normal primary astrocytes. We found that 154 proteins are downregulated and 101 upregulated in the clone. Moreover, 46 proteins are only expressed in the clone and 82 only in the normal cells. Notably, only 11 upregulated/unique proteins are encoded in the duplicated q arm of isochromosome 8 (i(8q)), which cytogenetically characterizes the clone. Since both normal and transformed brain cells release extracellular vesicles (EVs), which might induce epigenetic modifications in the neighboring cells, we also compared EVs released from transformed and normal astrocytes. Interestingly, we found that the clone releases EVs containing proteins, such as matrix metalloproteinase 3 (MMP3), that can modify the extracellular matrix, thus allowing invasion.

## 1. Introduction

Gliomas are heterogeneous brain cancers that derive from glial cells [1,2,3,4,5,6,7]. From the histological point of view, they maintain some properties of the cells from which they derive; thus, over time, they have been subdivided into astrocytomas, oligodendrogliomas, ependymomas and glioastrocytomas [1,8,9]. More recently, however, genetic and biochemical properties have been also studied as they give more precise indications on the type and grade of the tumor, also suggesting possible therapeutic approaches. Indeed, although recently brain cancer classification based on tumor histology has been potentiated, it has been also realized that it is not sufficient and that molecular data are also essential. On the basis of next-generation sequencing, it has been found, for example, that sometimes a few gene mutations can distinguish histologically identical brain tumors [10,11,12,13,14]. It is worth noting that these small differences can affect clinical progression and patient survival rates [7]. Thus, an integrated diagnosis, based on both classical and molecular criteria, is now highly recommended [14].

On the other hand, new therapies are also required because gliomas, especially the high-grade ones, are still fatal, in spite of well-defined therapies based on surgery, radiotherapy and chemotherapy [15,16,17], and, more recently, proton radiotherapy [18,19]. In particular, in order to develop new methodologies to deal with brain cancer therapy, it is mandatory to understand at best the molecular processes that induce glial cells/glial cell precursors to acquire invasive phenotypes [20,21]. With this in mind, we previously developed three astrocyte cell lines with increasingly transformed properties [22] and analyzed them at both the cytogenetic and epigenetic levels. We found that the most modified cell line (A-FC6) showed epigenetic and chromosomal alterations typical of tumor cells, even if, surprisingly, it expressed the differentiation-specific H1.0 linker histone variant at higher levels with respect to normal primary astrocytes [22]. In particular, the A-FC6 clone contains an isochromosome 8 (i(8q)), a chromosome aberration causing the q arm of chromosome 8 and consequently all the genes located therein to be duplicated.

Thus, in the present study, we decided to perform a proteomic comparison between the A-FC6 cell line and normal primary astrocytes, in order to detect molecular pathways that can be affected by transformed phenotype. We also focused on proteins encoded on the partially duplicated chromosome 8. Moreover, we analyzed the extracellular vesicles (EVs) released by both normal and transformed astrocytes. It is, indeed, now widely accepted that both normal and cancer cells are able to release EVs through which they transfer a variety of molecules (proteins, different classes of RNAs and lipids) to other cells, thus inducing epigenetic modifications of their behavior [23]. It has been also found that, in general, cancer cells produce many more EVs than normal cells, and that this ability is related to their capacity to transfer cancer properties to other cells while repressing the immune cell ability to recognize and fight them [5]. Herein, we report that, indeed, EVs released from the A-FC6 cell line contain proteases able to modify the extracellular matrix, a prerequisite for invading the brain parenchyma.

## 2. Materials and Methods

### 2.1. Cell Purification and Culturing

The experiments described in the present paper have been performed on normal astrocytes and on the A-FC6 clone that had been stored as frozen material; normal astrocytes had been purified from cortices of rat fetuses and the astrocyte clone had been established as previously described [22]. Those experiments had been approved by the Animal Welfare Committee of the University of Palermo, and authorized by the Ministry of Health (Rome, Italy; authorization number 69636.N.GCQ).

### 2.2. Proteomic Studies

When the cells reached sub-confluence, the Fetal Calf Serum (FCS)-containing medium was substituted with FCS-free medium, and cells were cultured for 24 h more. The medium was then removed, and three PBS washes were performed. The cells were detached with a scraper and suspended in PBS. The cell suspension was then centrifuged at 500× *g* for 5 min and the cell pellet was resuspended in an appropriate volume of “Nuclei Buffer” (NB: 0.32 m sucrose; 50 mm sodium phosphate buffer, pH 6.5; 50 mm KCl, 0.15 mm spermine; 0.15 mm spermidine; 2 mm EDTA; and 0.15 mm EGTA) containing protease inhibitors (2 μg/mL aprotinin, 2 μg/mL antipain, 2 μg/mL leupeptin, 2 μg/mL pepstatin A, 1.0 mM benzamidine and 1.0 mM phenylmethylsulfonyl fluoride, all from Merck Italy, Sigma-Aldrich). The cells were then homogenized with a Dounce homogenizer in an ice bath and stored at −80 °C until use. Protein concentration was determined by the Bradford protein assay, and aliquots containing 20 µg of total protein were used in triplicate for proteomic analyses. The total proteins were alkylated, reduced and digested using trypsin, as previously described [24]. The peptides obtained were desalted through a C18 chromatographic column, separated by liquid chromatography and identified by mass spectrometry. The nano-LC-MS/MS system included a nano-LC (EASY-nLC 1000, Proxeon—part of Thermo Scientific, Waltham, MA, USA) with an Acclaim PEPMap C18 column (50 cm × 75 µm ID, Thermo Scientific, Waltham), coupled online via a nano-spray flex ion source equipped with a PRSO-V1 column oven (Sonation, Biberach, Germany) to a Q-Exactive mass spectrometer (Thermo Scientific, Waltham, MA, USA). Peptides were separated with reverse-phase chromatography, using a 180 min binary gradient of water (A) and acetonitrile (B) containing 0.1% formic acid at 50 °C column temperature.

The mass spectra obtained were used for protein identification, using Maxquant software (maxquant.org, Max-Planck Institute Munich, version 1.5.2.6), searched against *Rattus norvegicus* reference database from UniProt. Trypsin was defined as protease and two missed cleavages were allowed for the database search. The option first search was used to recalibrate the peptide masses within a window of 20 ppm. For the main search, peptide and peptide fragment mass tolerances were set to 4.5 and 20 ppm, respectively. Carbamidomethylation of cysteine was defined as a static modification. Protein acetylation at the N-terminus and oxidation of methionine were set as variable modifications. A protein was considered identified if it was present in all three replicates analyzed.

### 2.3. Statistics

The Excel file obtained, containing the number of total proteins identified and their relative abundance, determined by means of the label-free quantification (LFQ) parameter, calculated by the analysis software based on the intensity of the mass spectra and “*p*-value”, was used for subsequent analyses.

### 2.4. Purification of EVs

Astrocytes were cultured in Dulbecco Modified Eagle Medium/Ham’s F12 (DMEM/F12) (2:1), until sub-confluence; cells were then washed twice in phosphate-buffered saline (PBS), pH 7.5, and incubated with FCS-free medium for 24 h. Conditioned media were centrifuged at 2000× *g* for 10 min and then at 4000× *g* for 15 min. The supernatant was centrifuged at 105,000× *g* (Ti60 Rotor, Beckman Coulter, Brea, CA, USA) for 90 min at 4°C. The pellet containing microvesicles (MVs) was resuspended in PBS, and protein concentration was determined using a Qubit Protein assay kit (Q33211, Thermo Fisher Scientific, Waltham, MA, USA).

### 2.5. Western Blot Analysis

Twenty-microgram samples of total proteins, prepared as described above, were loaded on 10% polyacrylamide-SDS denaturing gels. After electrophoresis, samples were blotted onto polyvinylidene difluoride (PVDF) membranes (0.45 µm pore-size, Amersham Biosciences, Little Chalfont, United Kingdom). Transfer of proteins to the membrane and concentrations of the samples were visualized by staining the membranes with Ponceau red for 10 min. Finally, membranes were immunostained with a mouse monoclonal anti-MMP3 antibody (anti-MMP-3 (Ab-1) Mouse mAb (55-2A4) IM36 from Calbiochem, CA, USA). The secondary anti-mouse antibody was from Promega (St. Louis, MO, USA).

## 3. Results

### 3.1. Comparative Proteomic Analysis

**Figure 1:**
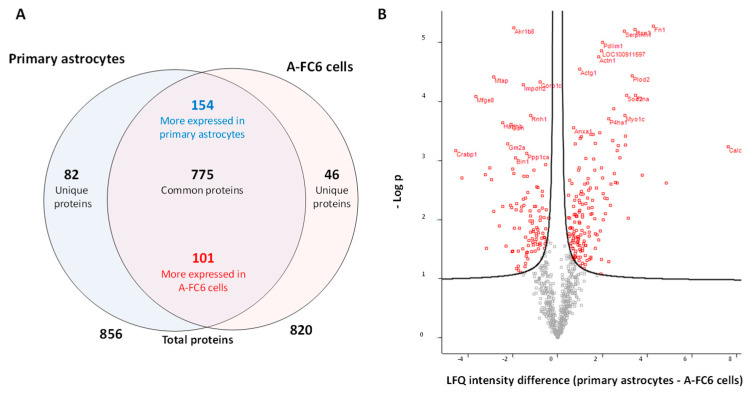
Comparative proteomic analysis of cell lysates from primary astrocytes and A-FC6 cells. (**A**) Venn diagram showing the proteins (unique, common, up/downregulated and total) identified in primary astrocytes and A-FC6 cells. (**B**) Volcano plot of significant proteins differentially expressed between primary astrocytes and the A-FC6. On the *X* axis, the differences in the intensity of “label-free quantification” (LFQ) are reported, for the proteins identified in primary astrocytes and A-FC6 cells; on the *Y* axis, the log of the *p*-value, corresponding to significance, is shown.

Comparative proteomic analysis of proteins extracted from the A-FC6 clone versus those from primary astrocytes identified a total of 903 proteins (Supplementary Table S1). As shown in the Venn diagram (Figure 1A), 775 proteins are expressed by both cell lines: among these, 101 and 154 are respectively upregulated and downregulated in A-FC6 with respect to primary astrocytes, while the expression of 520 proteins remains unchanged between the two cell lines (value obtained by subtracting the proteins whose regulation is varied from those in common in both cell lines). Furthermore, 82 proteins were identified exclusively in primary astrocytes, while 46 were identified exclusively in A-FC6, suggesting a significant modulation of the proteomic expression, probably responsible for the acquisition of a tumoral phenotype, including genomic instability. The quantitative differences between the identified proteins are shown in a volcano plot (Figure 1B), that reports, on the abscissa, the differences in the intensity of “label-free quantification” (LFQ), a parameter that quantitatively determines the expression of the protein under examination, of the proteins identified in the primary astrocytes and in A-FC6 cells, while the log of the *p*-value is reported on the ordinate.

**Figure 2:**
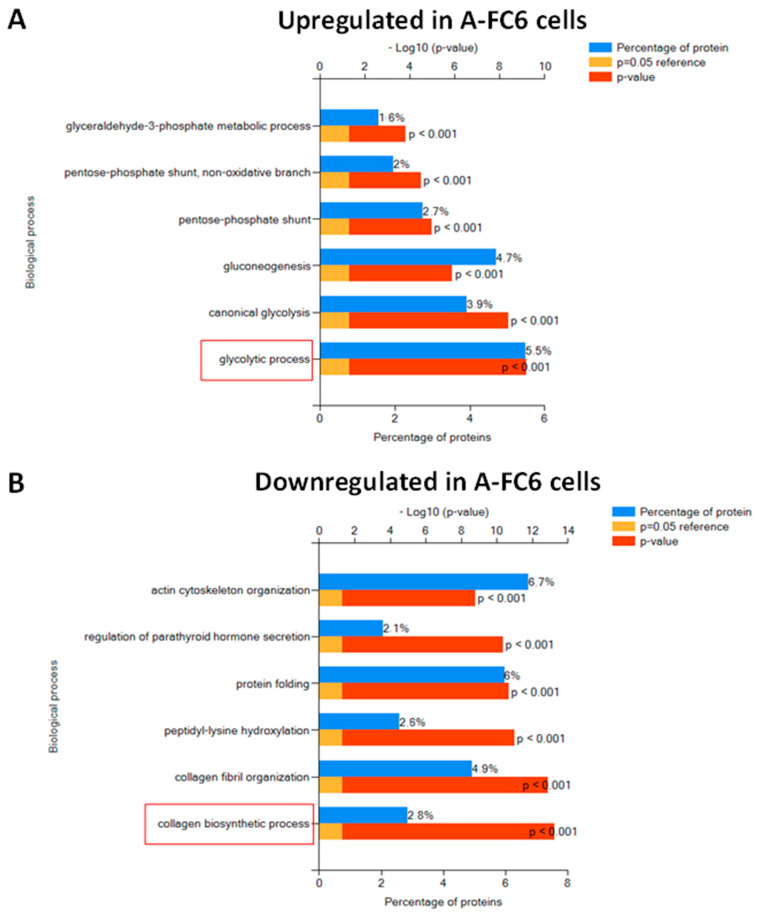
Enrichment pathway analysis of differentially expressed proteins in A-FC6. (**A**) Proteins upregulated in A-FC6. (**B**) Proteins downregulated in A-FC6. Significantly enriched biological pathways were ranked by *p*-value using the FunRich 3.0 software. Red boxes highlight significant pathways in which identified proteins are involved.

The 101 upregulated and the 46 uniquely expressed proteins evidenced in A-FC6 cells, as well as the 154 proteins downregulated in A-FC6 and the 82 uniquely expressed proteins of primary astrocytes, were highlighted by using the FunRich tool (Figure 2). Interestingly, the differentially expressed proteins were involved in different biological functions: while the upregulated proteins in A-FC6 clone are significantly involved in the glycolytic pathway (Figure 2A), proteins downregulated in the A-FC6 clone are significantly involved in the collagen biosynthetic pathway (Figure 2B). These biological properties are in accordance with the malignant phenotype shown by the A-FC6 clone. It is well recognized indeed that cancer cells reprogram their metabolism to support uncontrolled proliferation and metastatic progression, and that glycolysis is highly upregulated in solid tumors, which show increased anaerobic metabolism compared to aerobic respiration, which is generally downregulated. The shift of tumor metabolism, called the “Warburg effect”, plays a functional role in the acquisition of an invasive phenotype. On the other hand, it is now accepted that signals from the microenvironment influence cancer cell behavior and that the tumor microenvironment, including extracellular matrix (ECM) composition, largely influences tumor progression. In particular, the deregulation of the collagen biosynthetic pathway may promote cancer cell invasiveness. Collagen is degraded in the tumor microenvironment through the activity of the metalloproteinases and collagenases, generally overexpressed in aggressive tumors.

### 3.2. Comparative Proteomic Analysis with Respect to Chromosome #8 Gene Expression

**Figure 3:**
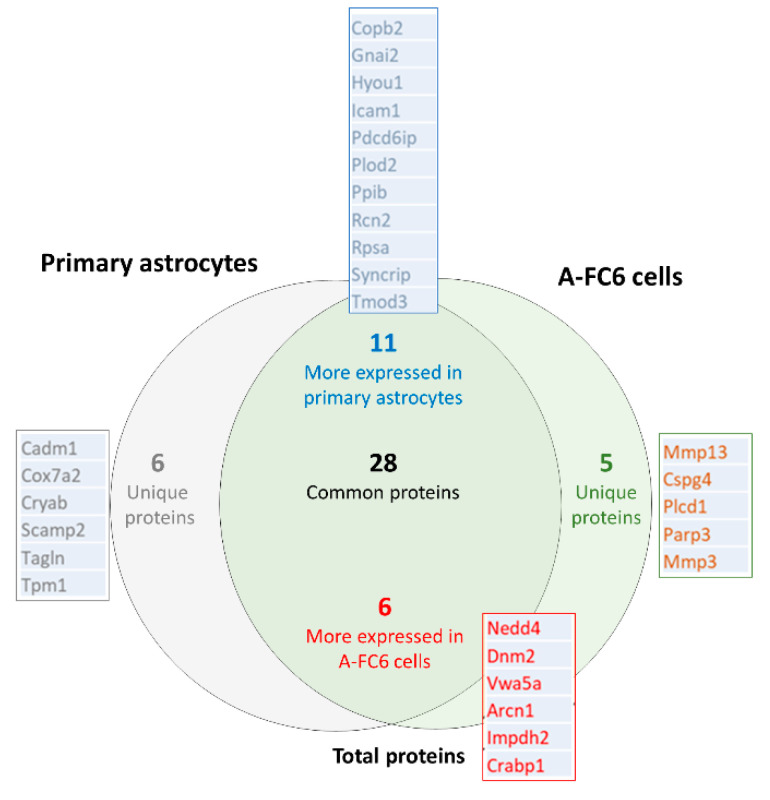
Venn diagram showing proteins expressed by chromosome #8 genes and identified in primary astrocytes and in A-FC6.

The *R. norvegicus* genome contains 43,258 genes and encodes 68,976 proteins. In particular, chromosome 8 contains 2222 genes and encodes 6% of all proteins (Wellcome Sanger Institute-NCBI, Cambridge, UK). Since the cytogenetic analysis of the A-FC6 clone showed the presence, in 100% of the observed metaphases, of an isochromosome 8 (i(8q)), due to duplication of the long arm of chromosome 8, we verified whether the expression of the genes present in chromosome 8 was significantly different between primary astrocytes and the A-FC6 clone. Surprisingly, no significant variation in the amount of proteins encoded by chromosome 8 was found when the two astrocyte cell populations were compared, suggesting that some compensatory mechanism of gene expression might be activated in cancer cells. However, as shown in the Venn diagram reported in Figure 3, among the 56 identified proteins that are encoded on chromosome 8, 28 remained unchanged,11 are more expressed in primary astrocytes and 6 are more expressed in the A-FC6 clone. At the same time, six proteins are expressed only in the primary astrocytes, while five proteins are expressed only in A-FC6 cells. Interestingly, two MMPs, namely MMP3 and MMP13, were more expressed in the A-FC6 clone, suggesting a possible link with the deregulation of the collagen degradation pathway.

### 3.3. Comparative Proteomic Analysis of Extracellular Vesicles (EVs) Released from Either Normal or Transformed Astrocytes

**Figure 4:**
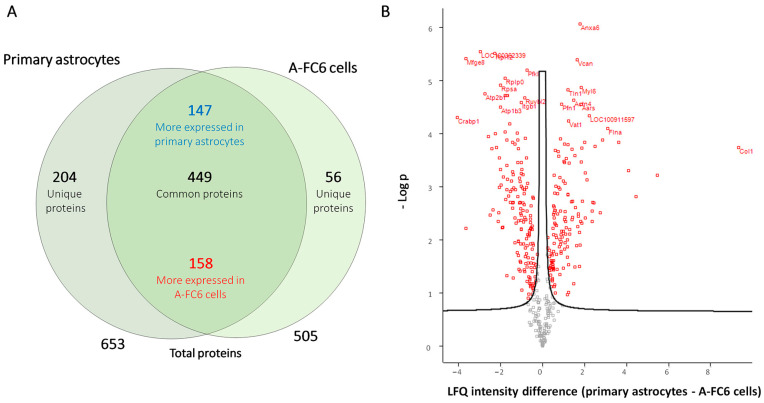
Comparative proteomic analysis of EVs from primary astrocytes and the A-FC6 cells. (**A**) Venn diagram showing the identified proteins (unique, common, up/downregulated and total) from primary astrocytes and the A-FC6 clone. (**B**) Volcano plot of significant proteins differentially expressed in primary astrocytes and A-FC6 cells. On the *X* axis, the differences in the intensity of “label-free quantification” (LFQ) of the proteins identified in primary astrocytes and A-FC6 cells are reported; on the *Y* axis, the log of the *p*-value, corresponding to significance, is reported.

**Figure 5:**
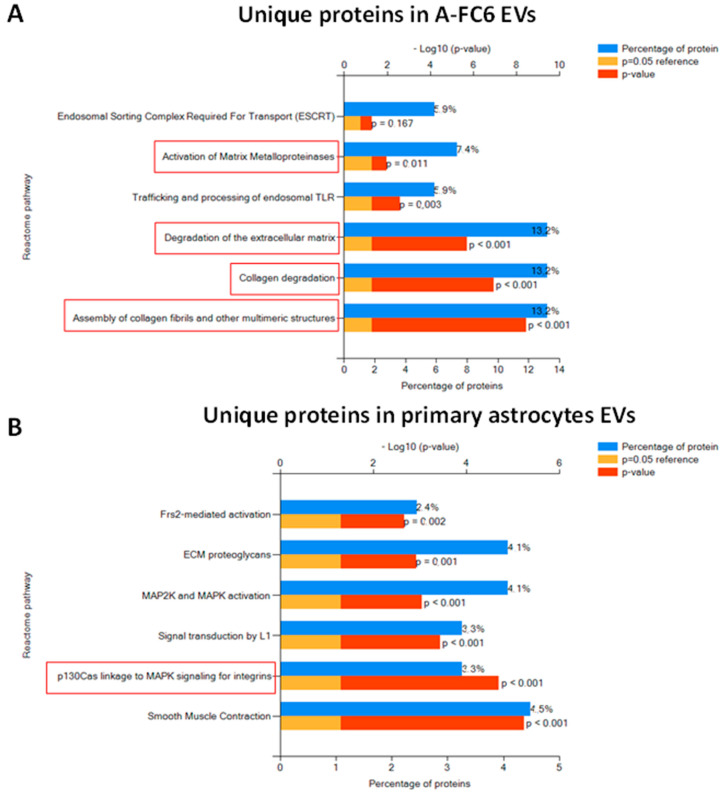
Enrichment pathway analysis of differentially expressed proteins in A-FC6. (**A**) Unique proteins identified in A-FC6-derived EVs. (**B**) Unique proteins identified in EVs derived from primary astrocytes. Significantly enriched pathways were ranked by *p*-value using the FunRich 3.0 software. Red boxes highlight the most significant pathways in which identified proteins are involved.

The comparative proteomic analysis was also performed on the extracellular vesicles (EVs) released from primary astrocytes and A-FC6 cells. Collectively, a total of 709 proteins were identified (Supplementary Table S2). Interestingly, among the list of the top 100 proteins commonly identified in exosomes (data derived from ExoCarta), 78 proteins were also identified in our experiments, confirming the good quality of our preparations. As shown in the Venn diagram (Figure 4A), 449 proteins are identified in all EVs: among these, 158 and 147 are respectively upregulated and downregulated in EVs released from A-FC6 cells with respect to EVs from primary astrocytes. Moreover, 204 proteins were identified exclusively in the EVs released from primary astrocytes, while 56 were identified exclusively in the EVs released from the A-FC6 clone. The quantitative differences between the identified proteins are shown in a volcano plot (Figure 4B). The biological pathway enrichments performed on the proteins uniquely identified in EVs derived from the A-FC6 clone and primary astrocytes (Figure 5) confirmed that the specific cargo of EVs is indicative of cancer cell properties: In accordance with previous findings, proteins present only in A-FC6-derived EVs were involved in the ECM degradation, and collagen degradation, in particular. Proteins present only in primary astrocyte-derived EVs were involved in cell–matrix interaction.

Interestingly, eight of the proteins uniquely identified in EVs derived from A-FC6 clone, namely Integrin Subunit α 8 (Itga8), Mmp13, Serpine2, Phospholipase C Delta 1 (Plcd1), Embigin (Emb), Mmp3, Complement defense (Cd59), and Polyunsaturated fatty acid lipoxygenase (Alox15), were also identified as unique in A-FC6 cell lysate.

**Figure 6:**
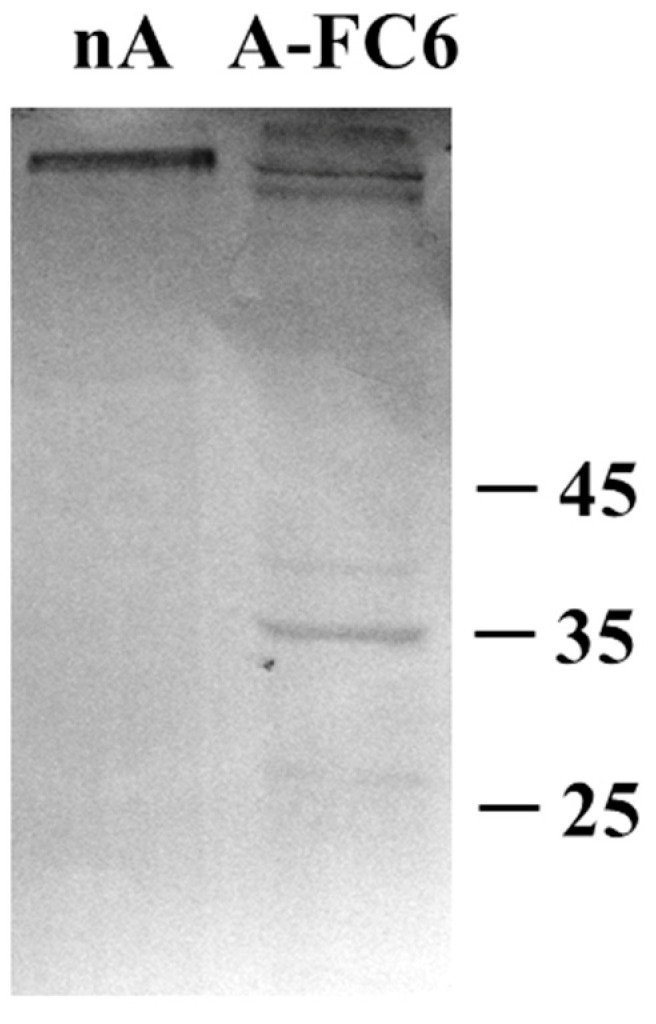
Western blot analysis of proteins present in EVs released from normal astrocytes (nA) and A-FC6 cells. After blotting, the membrane was immunostained with a mouse monoclonal anti-MMP3 antibody (Calbiochem, CA, USA). The secondary anti-mouse antibody was from Promega (St. Louis, MO, USA).

As a confirmation of the data reported above, we analyzed EVs from both primary astrocytes and A-FC6 cells by Western blot analysis (Figure 6) with antibodies directed against MMP-3, an extracellular matrix metalloproteinase, which can have an important effect on the capacity of cells to invade brain parenchyma. Interestingly, we only observed proteins recognized by antibodies in EVs from the A-FC6 clone. The sizes of these bands suggest that MMP3 is probably included in EVs in activated forms, which are indeed shorter than the inactive pro-MMP3 (expected to be about 52–54 kDa).

## 4. Discussion

The fundamental role of astrocytes in brain functions has been recognized for many years. First of all, they give metabolic support to neurons; they contain, for example, glycogen that is used when a special expense of energy is required for neuronal functions, as occurs during learning and memory processing [25,26,27]. In these conditions, it has been found that lactate, produced at the end of glycolysis by astrocytes, can be released from these cells and taken up by neurons that can oxidize it to pyruvate in order to produce acetyl-CoA, immediately used in the tricarboxylic acid cycle [28]. In addition, astrocytes are also able to control extracellular levels of ions and neurotransmitters, such as glutamate: in this case, astrocyte function has both a metabolic effect and a role in limiting glutamatergic spillover at extra-synaptic sites [29]. More recently, it is becoming increasingly clear that additional and more complex roles are also played by astrocytes. These cells are indeed able to respond to neurotransmitters and are surprisingly also capable of releasing in turn signaling molecules, now called gliotransmitters [30,31,32].

Last but not least, astrocytes normally form a network, able to influence at the same time a great number of different synapses [32,33].

Now, the most common brain cancers derive from glial cells, especially from astrocytes; these tumors are very aggressive and show a high mitotic index [5]. In addition, all the fundamental roles played by normal astrocytes, as mentioned above, can be lost or at least altered when these cells are transformed.

Our research study aimed at contributing to the understanding of some dysfunctional alterations of astrocytes (which are thought to be responsible for the genesis of most brain tumors) through the isolation of astroglial cell lines from *R. norvegicus* embryos.

The general aim was to obtain an in vitro model, composed of various astrocyte cell lines [22], with an increasing degree of genomic instability that could reproduce the analogous genesis of human central nervous system (CNS) tumors, thus hopefully contributing to a better understanding of the entire process and to identifying indications for new therapeutic approaches.

### 4.1. Proteomic Analysis

We analyzed, with a proteomic approach, the A-FC6 cells, the most unstable of the previously obtained astrocyte clones [22], with the initial aim of studying in particular the proteins encoded by the genes present in chromosome i(8q), the principal characterizing aberration that we previously found in this clone. Proteomic data (Figure 1 and Figure 2) showed a qualitative/quantitative modulation between primary astrocytes and A-FC6 cells. In detail, 82 proteins were identified only in primary astrocytes and 46 only in A-FC6; on the other hand, 154 and 101 were more expressed in primary astrocytes and in the A-FC6 clone, respectively. Furthermore, taking into account the chromosomal localization of the identified proteins, no significant upregulation of the proteins encoded by genes present on chromosome 8 in the A-FC6 cells was recorded, despite the presence in the clone of an isochromosome 8q. Specifically, among the 56 identified proteins encoded by chromosome 8, 11 are more expressed in primary astrocytes and 6 are more expressed in the A-FC6 clone. At the same time, six proteins are expressed only in the primary astrocytes, while five proteins are expressed only in A-FC6 cells.

Thus, qualitative and quantitative proteomic analyses performed on primary astrocytes and A-FC6 cells suggested that the presence of the supernumerary genes in i(8q) does not result in their widespread increase in protein expression. In other words, from a proteomic point of view, it does not seem that there has been an appreciable upheaval, compared to primary astrocytes, in spite of the exclusive presence of some proteins (Figure 6), such as the following:

- The metalloproteinases Mmp3 and Mmp13, which are known to be involved in tumor invasion processes, since these proteases can degrade the extracellular matrix and also intracellular polymers, causing inflammation [34,35]. This suggests that the genomically more unstable line could favor a higher permeability of the brain parenchyma;
- Chondroitin sulfate proteoglycan 4 (Cspg4), involved in the cell–substrate interaction of the endothelium and in the maturation of oligodendrocytes [36,37];
- Phospholipase C-delta1 (Pcld1), involved in cell cycle control [38];
- Poly (ADP-ribose) polymerase 3 (Parp3), involved in different transcriptional regulatory pathways in neuronal development [39].

Some specific upregulated proteins were also identified (Figure 5), such as the following:

- The E3 ubiquitin protein ligase (Nedd4), which, according to what is reported in the literature, is involved in the growth and development of neurons [40,41];
- Dynamin 2 (Dnm2), which participates in pre- and post-synaptic vesicular trafficking and endocytosis [42,43];
- Von Willebrand factor (Vwa5a), a glycoprotein secreted by the endothelium and involved in some processes, such as platelet adhesion, development of the perivascular matrix and alteration of calcium signaling, glutamate absorption and the activity of some metalloproteinases [44];
- Archain 1 (Arcn1), which directs the maturation of the cerebellum and the intracellular vesicular traffic and seems to play a direct role also in neurodegenerative diseases [45];
- Inosine monophosphate dehydrogenase-2 (Impdh2), involved in the biosynthesis of GTP and in the support of cell proliferation [46];
- Cellular retinoic acid binding protein 1 (Crabp1), which plays a key role in various phosphorylation pathways, cell proliferation and CNS regulation events [47,48].

### 4.2. EVs

It is now widely recognized that extracellular vesicles constitute a form of universal and powerful communication that allows cells, from prokaryotes to the most complex eukaryotes, to exchange information. EVs indeed transport a variety of proteins, as well as coding and non-coding RNAs, lipids and metabolites, that can induce epigenetic adaptations in the surrounding receiving cells [49].

In the mammalian brain, it is now clear that cell-to-cell communication mediated by EVs is of the highest importance for the regulation of differentiation of all the brain cell types, from neurons to astrocytes, endothelial cells, oligodendrocytes and microglia, and especially for the formation of synaptic contacts [23]. EVs have been also reported to be essential for the establishment and maintenance of the blood–brain barrier (BBB) [50]. EVs released from both neurons and astrocytes contain indeed two angiogenic factors, namely fibroblast growth factor 2 (FGF2), and vascular endothelial growth factor 2 (VEGF2) [51,52], and are thus probably responsible for inducing the brain capillary endothelial cells (BCECs) to acquire the specific phenotype that allows the formation of the BBB.

Although EVs are fundamental vehicles for normal cell-to-cell communication in the brain, it is now clear that the ability to produce EVs can become a problem when the cells assume pathological phenotypes, as occurs in neurodegeneration or when they turn into cancer cells.

The release of EVs is particularly intense in tumor cells, which use EVs and their content to escape immune surveillance and to create an environment prone to invasion [5].

Herein, we report that EVs released from A-FC6 cells indeed contain proteins able to contribute to these processes. Among them, for example, are the following: (i) Cd59, a membrane complement regulatory protein that has been demonstrated to be overexpressed in most solid tumors, where it facilitates tumor cell escape from complement surveillance; (ii) integrins, a unique class of signaling molecules that are implicated in cancer progression as mechanotransducers and as key components of the cell migration machinery, the alterations of the expression patterns of which have been observed in most malignant tumors; (iii) matrix metalloproteinases, such as MMP3, which are able to modify the extracellular matrix, thus allowing tumor cell migration.

## 5. Conclusions

In conclusion, our proteomic studies showed some functional proteomic modulation between the two cell types which is useful for the acquisition of malignant characteristics by the A-FC6 clone. However, although 100% of A-FC6 cells possess the cytogenetic marker i(8q), which increases the number of copies of the genes located on the q arm of chromosome 8, the proteins encoded by these genes, identified by Western blotting, are not consequently upregulated, suggesting a compensation mechanism at the level of protein expression due to post-transcriptional mechanisms.

Most importantly, we found that EVs released from the transformed cells contain proteins that can facilitate escape from immune surveillance and that can allow remodeling of the extracellular matrix and cell invasion.

## Data Availability

The results of the reported experiments are all described in the text. The Authors are anyway available for any question and further explanations.

## Supplementary Materials

The following supporting information can be downloaded at: https://www.mdpi.com/article/10.3390/genes14050990/s1
